# Surgically Relevant Morphological Parameters of Proximal Human Femur: A Statistical Analysis Based on 3D Reconstruction of CT Data

**DOI:** 10.1111/os.12416

**Published:** 2019-02-27

**Authors:** Ehsan Soodmand, Guoyan Zheng, Wolfram Steens, Rainer Bader, Lutz Nolte, Daniel Kluess

**Affiliations:** ^1^ Department of Orthopaedics University Medicine of Rostock Rostock Germany; ^2^ Institute for Surgical Technology and Biomechanics University of Bern Bern Switzerland

**Keywords:** Femur morphology, Hip joint, Impingement, Morphological study, Proximal femur

## Abstract

**Objectives:**

Recently, more accurate description of the femoral geometry has become of interest to engineers and orthopedic surgeons. However, an appropriate database is lacking. Therefore, the aim of this study is to present morphological parameters and their correlations, which are relevant for medical issues such as impingement after total hip replacement, as well as for implant design and the etiology of hip fractures.

**Methods:**

We investigated 12 well‐known morphological parameters of the femur in 169 healthy human subjects through evaluation of 3D‐reconstructed CT scans. Pearson's coefficients of correlations were calculated using a statistical *t*‐test method for each pair of parameters.

**Results:**

The mean, maximum, minimum, median, and standard deviation values are reported for all parameters. Histograms showing the distribution of each morphological parameter are also presented. It is shown that absolute and horizontal offsets, total femur length, and NCVD parameters are normally distributed, but NCDF and NCDS are not. Furthermore, an inter‐correlation matrix was reported to reveal statistical correlations between these parameters. The strongest positive correlation existed between absolute offset (OSA) and horizontal offset (OSH), while the least positive correlation was found between NCDF and total femur length (TFL), and also between NCDS and NCDF. Anteversion angle (ATA) and OSA showed the least negative correlation. However, the strongest negative correlation was found between neck‐shaft angle (NSA) and greater trochanter height (GTH), as well as between OSA and NCVD.

**Conclusions:**

Comprehending patients’ native bone morphology, including the variations and correlations, is essential for orthopedic surgeons to undertake preoperative planning and surgery as well as to appropriately design medical devices. Thus, more population‐based detailed databases are necessary. We investigated an extensive set of proximal femoral morphology parameters using a statistically standardized method to expand the existing knowledge. The results of our study can be used for diverse medical and biomechanical purposes.

## Introduction

The human femur, as a bone connected to both the hip and the knee joint, plays a key role in the biomechanics of gait and posture. The morphology of the femur is directly connected to biomechanical factors which have clinical impact. For example, the length of the femoral neck determines the lever arm of muscles attached to the greater trochanter with respect to the center of rotation of the hip joint and is connected to femoral offset^1^. Disregarding the femoral offset was shown to result in limited functional outcome and pain following total hip replacement (THR)^2^. Abnormal morphology, such as joint deformity, has been observed to affect the development of early osteoarthritis (e.g. in cases of high neck‐shaft angle [NSA])^3^. Other morphological parameters, such as the femoral head diameter (FHD) and the anteversion angle (ATA), influence the range of motion (ROM) of the hip joint. Hence, femoral investigation has driven the desire of biomechanists and orthopedic surgeons to enhance the current methods.

Orthopedic surgeons performing THR must be aware of the biomechanical behavior and the physiological function of the hip joint while undertaking preoperative planning^4^, ^5^. Orthopedic surgeons adapt the neck shaft angle, the vertical offset (OSV), and the horizontal offset (OSH) through selection of type, size, and model of total hip stem and head. In contrast, for hip resurfacing arthroplasty (HRA), where only the cartilage surface is replaced by a metal cap, the surgeon needs to deal with the given morphology of the femur and has only a minor influence on the relevant parameters. Due to the limited availability of published data, more comprehensive knowledge of femoral morphology is crucial to deal with modern medical issues such as impingement after THR^6^ and HRA^7^, the etiology of hip fracture^8^, and proper implant design for orthopedic implants^9^, Postoperatively, bone–implant mismatch is the main reason for issues such as thigh pain, aseptic loosening, and impingement^6^. Oversized implants can intraoperatively provoke splintering, while screws that are too large may decancellate bone, leading to avascular necrosis; in addition, the thread may fail to fully cross the fracture site without providing sufficient compression for proper healing^10^. In addition, femoral offset and the head–neck ratio influence the joint ROM and the integrity of the artificial hip in relation to femoroacetabular impingement^11^. The outcome of THR is affected by the neck‐shaft angle (NSA). Better investigation of the NSA could help to effectively anticipate the incidence of femur fracture^12^, especially when osteoporotic^13^.

Assessment of the anteversion angle (ATA) of the femoral neck is also crucial for positioning of hip replacement^14^ and can reduce postoperative malrotation after intramedullary nailing of femoral shaft fractures^15^. In addition, ATA has a remarkable influence on the incidence of osteoarthritis and hip dysplasia^16^.

Analysis of abnormalities in femoral shape and orientation can help to predict degenerative diseases such as osteoarthritis^16^. Therefore, accurate descriptions of angles and bone dimensions are crucial to design nails, plates, and orthopedic prosthetics, as well as for preoperative planning^10^, ^15^. Therefore, over the past decade, establishment of a comprehensive overview of femoral morphological parameters has become a focus in hip joint‐related treatments such as hip resurfacing arthroplasty^17^. Several studies have been undertaken to investigate femoral geometrical parameters. However, few suggest a complete set of parameters including their correlation and surgical relevance. Thus, in the present study, we analyzed the femoral morphology using a reliable statistical approach of examining 12 essential morphological parameters of the proximal human femur. We provide a detailed discussion on the clinical relevance of these parameters. Because the diversity observed in femur morphology is challenging, orthopedic surgeons and engineers need a comprehensive overview for preoperative planning as well as to optimize prosthetic design. The data presented in this study can be used in different medical/biomedical research areas.

## Material and Methods

### *Specimens*

This study was conducted in collaboration with the Biomechanics and Implant Technology Research Laboratory of Orthopedics at University of Rostock (FORBIOMIT) and the Institute for Surgical Technology and Biomechanics at University of Bern (ISTB). 3D reconstruction of CT images, as a well‐established and precise method to carry out morphological studies, was used here^18^. The study was performed on 169 adult healthy femurs using CT scans obtained in supine position. CT images had a slice thickness of 0.625–1.5 mm and a pixel dimension of 0.652–1.087 mm. CT data of 129 femurs were acquired from the University of Bern and an additional 40 CT scans were provided by the Department of Anatomy of the University of Lübeck, Germany.

### *Reconstruction of 3D Models*

DICOM files of 169 CT scans were used to segment the surface of human femurs. At ISTB, a fully automatic method for segmentation of CT images of proximal human femur was applied using a combination of fast random forest regression‐based landmark detection and atlas‐based segmentation with an articulated statistical shape model instantiation^19^. The statistical shape model of the femur was constructed using an in‐house pipeline which combined surface‐based affine registration with intensity‐based non‐rigid registration to establish vertex‐to‐vertex correspondences between a randomly‐selected reference model and all remaining femoral models. To reduce the bias caused by reference model selection, the pipeline was executed twice where the average model obtained from the first execution was used as the reference model for the second execution. Principal component analysis was then applied to the aligned surface models to compute the statistical shape model. The constructed statistical shape model used in this study has also been successfully applied in a 2D−3D reconstruction application^20^, which demonstrated the validity of the constructed model. At FORBIOMIT, commercial software, AMIRA v.5.4.1 (Zuse Institute, Berlin, Germany), was used for reconstruction of a 3D model of human femurs from the CT images. The bony structures were labeled in all slices of the CT images based on the values of the Hounsfield unit (HU) for bones. Removal of the holes and sharp edges that were formed due to semi‐automated segmentation was undertaken in Amira using an established protocol^21^, ^22^ to reconstruct the surfaces accurately.

### *Parameters Definition*

**Figure 1:**
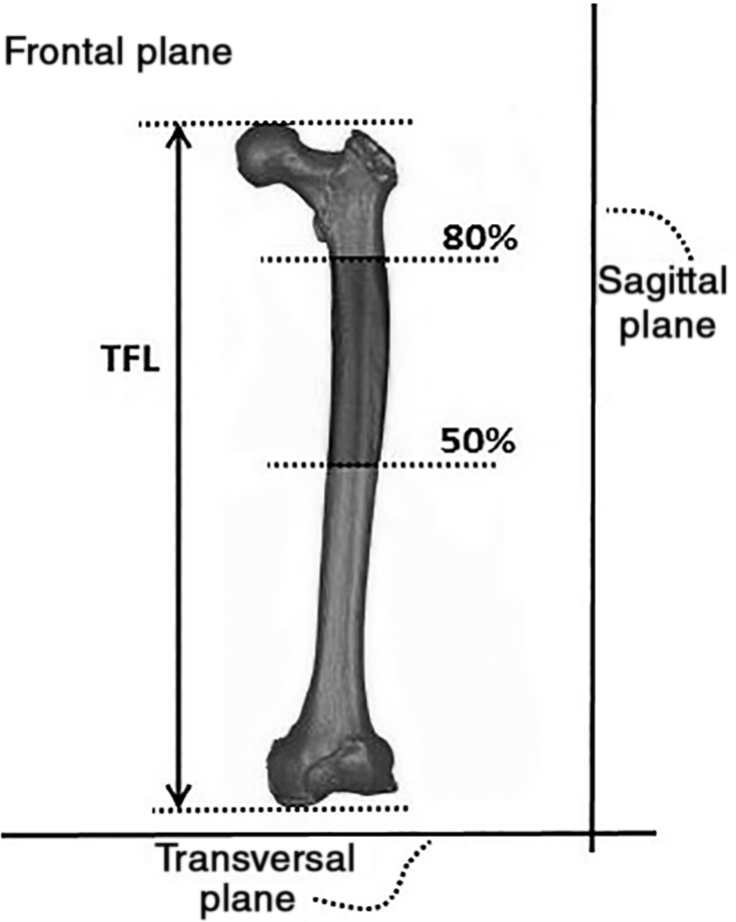
Defined frontal, sagittal, and transversal planes. 50% and 80% indicate half of the femoral length and 80% of femoral length from distal point respectively. TFL, total femoral length.

**Figure 2:**
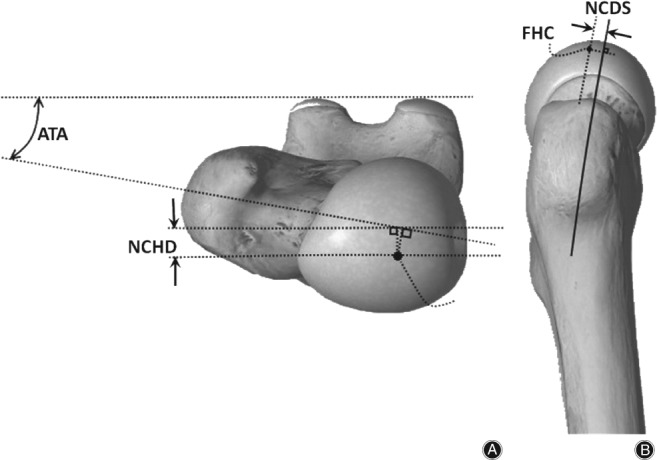
(A) Superior and (B) lateral view of human femur and illustration of some of the defined parameters as follows: ATA indicates anteversion angle; NCHD indicates the distance between the FHC and a plane parallel to the frontal plane containing the projection of the FHC to the FNA; FHC indicates femoral head center; NCDS shows the distance between FHC and femoral neck axis (FNA) projected to the sagittal plane. (The femur shape itself was reproduce using the freely available software called Essential Skeleton 4 [3D4Medical.com, LLC], but the defined parameters were indicated and drawn by the authors^34^.)

**Figure 3:**
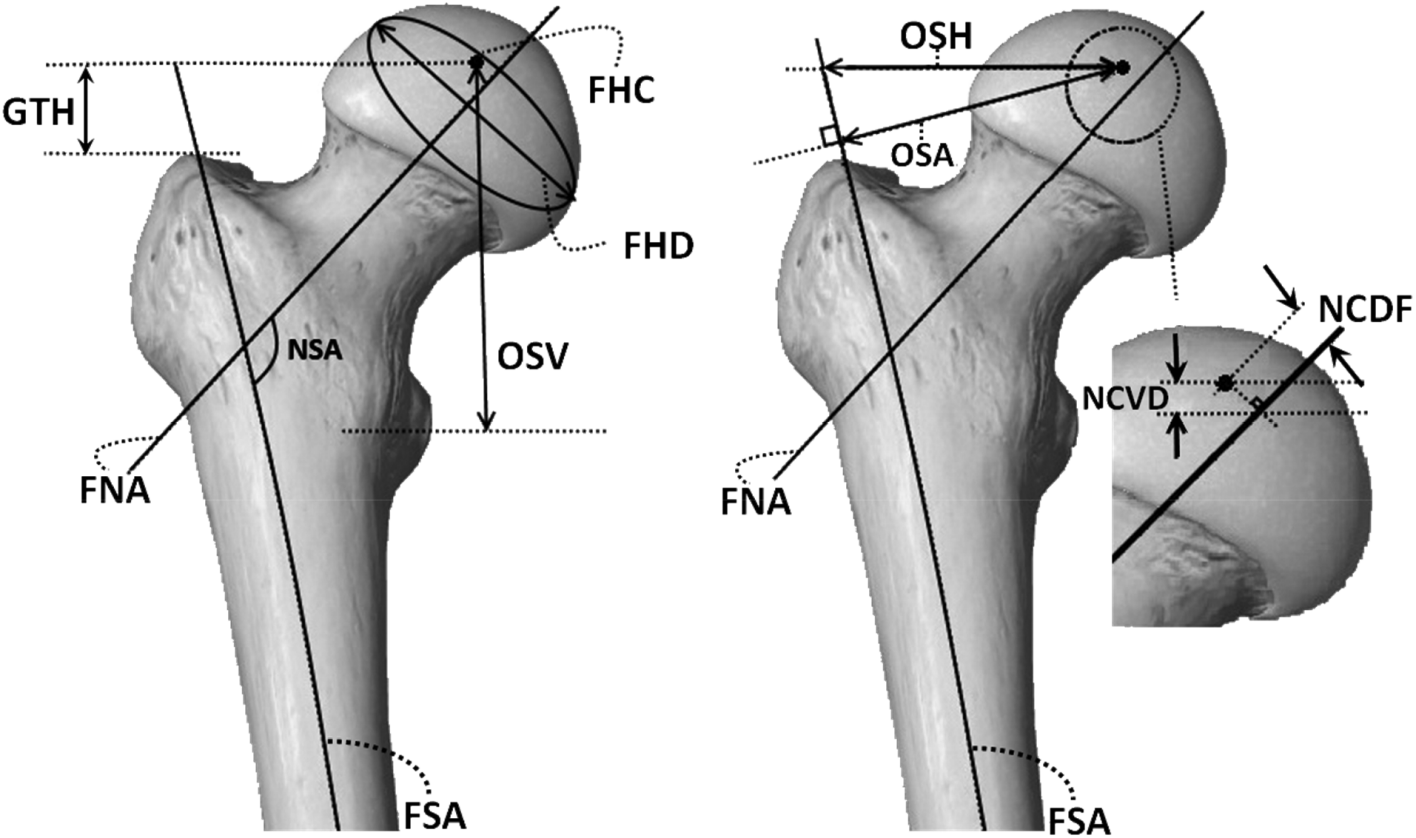
Anterior view of human femur demonstrating morphological parameters defined as follows: FHC, femoral head center; FHD, femoral head diameter; FNA, femoral neck axis; FSA, femoral shaft axis; GTH, greater trochanter height; NCDF, distance between the FHC and a plane parallel to the frontal plane containing the projection of the FHC to the FNA; NCVD, vertical distance between the FHC and a plane parallel to transversal plane containing the projection of the FHC to the FNA; NSA, neck‐shaft angle; OSA, absolute offset as the distance between femoral head center (FHC) and femoral shaft axis; OSH, horizontal offset as the projected distance between FHC and FSA to the frontal plane; OSV, vertical offset as the vertical distance between FHC and the plane parallel to transversal plane containing the center of the lesser trochanter. (The femur shape itself was reproduce using the freely available software called Essential Skeleton 4 [3D4Medical.com, LLC], but the defined parameters were indicated and drawn by the authors^34^.)

The frontal, sagittal, and transversal planes (Fig. 1) were defined on the 3D reconstructed models of femoral bones. Twelve descriptive parameters for the proximal femur were then selected as shown in Figs 2 and 3. The parameters are as follows:

1. Femoral head diameter (FHD). The diameter of best‐fit sphere of the femoral head irrespective of its vertical or transverse orientation.
2. Total femur length (TFL). Distance between the most distal point in the transversal plane and a parallel plane containing the most proximal point of the femur.
3. Neck‐shaft angle (NSA): Angle made by axis of femoral shaft and the line which passes through the center of the femoral head along the axis of the femoral neck.
4. Anteversion angle (ATA): Angle between a transverse line passing through the femoral head and neck center and an imaginary transverse line running medially to laterally through the knee joint.
5. Absolute offset (OSA): Distance between femoral head center (FHC) and femoral shaft axis (FSA). Femoral shaft axis was constructed by selecting the diaphysis part between 50% and 80% of the length of the femur (Fig. 1).
6. Vertical offset (OSV): Vertical distance between the FHC and the plane parallel to the transversal plane containing the center of the lesser trochanter.
7. Horizontal offset (OSH): Projected distance between FHC and FSA to the frontal plane.
8. Greater trochanter height (GTH): Vertical distance between FHC and the plane parallel to the transversal plane containing the most proximal point of the greater trochanter.
9. Distance between FHC and femoral neck axis (FNA) projected to the sagittal plane (NCDS).
10. Distance between FHC and FNA projected to the frontal plane (NCDF).
11. Vertical distance between the FHC and a plane parallel to transversal plane containing the projection of the FHC to the FNA (NCVD); positive for cranial positions of the FHC and negative for caudal positions.
12. Distance between the FHC and a plane parallel to the frontal plane containing the projection of the FHC to the FNA (NCHD); positive for anterior position of the FHC and negative for posterior position.

### *Parameter Measurement*

A statistical shape model^19^ was constructed from 129 models after establishing vertex by vertex correspondences at ISTB. The geometrical parameters of each femur were then obtained by propagating the associated measurements from the mean model of the statistical shape model to each individual femur. The morphological parameters for the remaining 40 femurs were measured at FORBIOMIT. The stereolithography (STL) files of the femurs were imported into Geomagic studio v.10 (Geomagic, Research Triangle Park, NC, USA) to obtain the morphological parameters of the proximal femur as described above^23^. Each parameter was measured six times and the average of all datasets were then used to decrease the probability of error.

### *Statistical Analysis*

The morphological data obtained from both the abovementioned methods were collected. Minimum (Min), maximum (Max), mean, median, and standard deviation (SD) were calculated in Microsoft Excel 2013. Pearson's coefficients of correlation were calculated using a statistical *t*‐test method in R v.3.2.2 (R Core Team, www.r-project.org) for each pair of morphological parameters. The significance level was set at *P* < 0.05.

## Results

**Figure 4:**
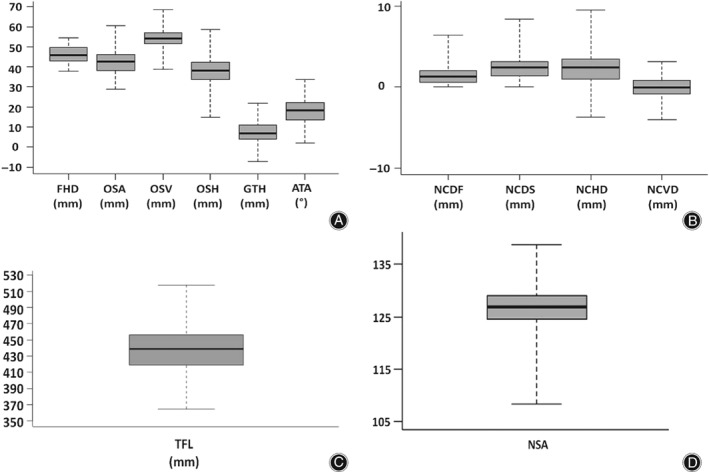
Box plots of morphological parameters’ distribution. (A) ATA, anteversion angle; FHD, femoral head diameter; GTH, greater trochanter height; OSA, absolute offset as the distance between femoral head center (FHC) and femoral shaft axis; OSH, horizontal offset as the projected distance between FHC and FSA to the frontal plane; OSV, vertical offset as the vertical distance between FHC and the plane parallel to transversal plane containing the center of the lesser trochanter. (B) NCDF, distance between the FHC and a plane parallel to the frontal plane containing the projection of the FHC to the FNA; NCDS, distance between FHC and femoral neck axis (FNA) projected to the sagittal plane; NCHD, distance between the FHC and a plane parallel to the frontal plane containing the projection of the FHC to the FNA; NCVD, vertical distance between the FHC and a plane parallel to transversal plane containing the projection of the FHC to the FNA. (C) TFL, total femur length. (D) NSA, neck‐shaft angle. All units are in mm except ATA and NSA, which are in degree.

The minimum, maximum, mean, SD, and median of the morphological parameters investigated in the current study are shown in Table 1. For instance, the maximum SD was observed in the measurement of TFL (29.62), while the minimum SD was found in the measurement of NCDF (1.22). The box plots shown in Fig. 4 display the mean, the minimum, and the maximum of the morphological parameters.

**Table 1:** Statistical calculation (minimum, maximum, mean, standard deviation (SD), and median) of the morphological parameter

Morphological parameters	Minimum	Maximum	Mean ± SD	Median
FHD (mm)	37.95	54.35	46.29 ± 4.02	45.82
OSA (mm)	28.79	60.52	42.39 ± 5.98	42.61
OSV (mm)	38.73	68.46	54.37 ± 5.14	54.23
OSH (mm)	14.88	58.60	37.90 ± 6.95	38.16
ATA (°)	1.99	33.57	17.46 ± 6.77	18.45
NSA (°)	108.37	138.72	126.35 ± 4.29	126.81
GTH (mm)	−7.38	21.75	7.44 ± 5.06	6.95
TFL (mm)	364.80	517.93	439.22 ± 29.62	438.89
NCDF (mm)	0.01	6.45	1.51 ± 1.22	1.30
NCDS (mm)	0.06	8.41	2.42 ± 1.38	2.40
NCVD (mm)	−4.03	3.18	−0.09 ± 1.43	−0.01
NCHD (mm)	−3.75	9.52	2.17 ± 1.90	2.40

ATA, anteversion angle; FHD, femoral head diameter; GTH, greater trochanter height; NCDF, distance between the FHC and a plane parallel to the frontal plane containing the projection of the FHC to the FNA; NCHD, distance between the FHC and a plane parallel to the frontal plane containing the projection of the FHC to the FNA; NCVD, vertical distance between the FHC and a plane parallel to transversal plane containing the projection of the FHC to the FNA; NCVD, vertical distance between the FHC and a plane parallel to transversal plane containing the projection of the FHC to the FNA; NSA, neck‐shaft angle; OSA, absolute offset as the distance between FHC and femoral shaft axis; OSH, horizontal offset; OSV, vertical offset; TFL, total femur length

**Figure 5:**
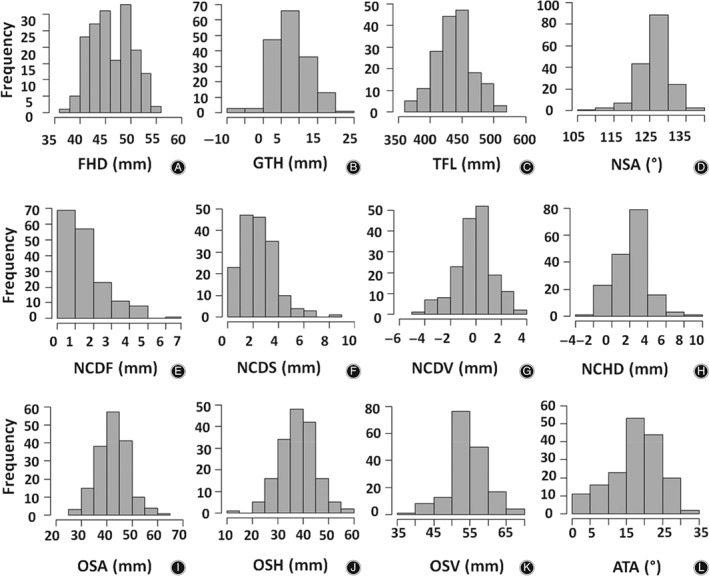
Histograms of the morphological parameters showing their distribution as follows: (A) FHD, femoral head diameter. (B) GTH, greater trochanter height. (C) TFL, total femur length. (D) NSA, neck‐shaft angle. (E) NCDF, distance between the FHC and a plane parallel to the frontal plane containing the projection of the FHC to the FNA. (F) NCDS, distance between FHC and femoral neck axis (FNA) projected to the sagittal plane. (G) NCVD, vertical distance between the FHC and a plane parallel to transversal plane containing the projection of the FHC to the FNA. (H) NCHD, distance between the FHC and a plane parallel to the frontal plane containing the projection of the FHC to the FNA. (I) OSA, absolute offset as the distance between femoral head center (FHC) and femoral shaft axis. (J) OSH, horizontal offset as the projected distance between FHC and FSA to the frontal plane. (K) OSV, vertical offset as the vertical distance between FHC and the plane parallel to transversal plane containing the center of the lesser trochanter. (L) ATA, anteversion angle. All units are in mm except ATA and NSA, which are in degree.

Figure 5 includes histograms showing the distribution of each parameter's diversity. For instance, this shows that NCDF and NCDS are not normally distributed. However, OSA, OSH, TFL and NCVD display an acceptable normal distribution.

Table 2 shows the inter‐correlation matrix which states the Pearson's correlation between any two morphological parameters. As shown here, there exist both positive and negative correlations between the parameters. Here, some strong correlations between some of the parameters are reported. TFL and FHD have a direct strong correlation (Pearson's coefficient of 0.68); therefore, a femur with a greater TFL also has a greater FHD. The OSA and the OSH show the strongest positive linear relationship (Pearson's coefficient of 0.92). The OSV has a strong positive correlation to the TFL (Pearson's coefficient of 0.75). A strong direct correlation was also found for NCDS and NCHD (Pearson's coefficient of 0.77). GTH and NSA as well as NCVD and OSA have the strongest inverse correlation (Pearson's coefficient of −0.62); thus, an increase in NSA is associated with a decrease in the distance between FHC and the great trochanter. The least negative correlation was found between ATA and OSA (Pearson's coefficient of −0.01). However, the least positive correlation was observed between NCDF and TFL and NCDS and NCDF (Pearson's coefficient of 0.03).

**Table 2:** The inter‐correlation matrix (*P* values) analyzed for the morphological parameters

Morphological parameters	TFL	NCVD	GTH	NCDF	OSA	NCDS	OSH	NSA	ATA	NCHD	OSV
FHD	0.68	−0.29	0.08	0.20	0.29	0.07	0.27	0.11	−0.09	0.08	0.52
TFL	‐	−0.03	−0.09	0.03	0.31	0.12	0.30	−0.05	−0.23	−0.03	0.75
NCVD	‐	‐	−0.55	−0.43	−0.62	0.27	−0.56	0.21	0.00	−0.05	0.25
GTH	‐	‐	‐	0.38	0.39	−0.18	0.41	−0.62	−0.02	−0.04	−0.54
NCDF	‐	‐	‐	‐	0.26	0.03	0.25	−0.07	0.06	0.11	−0.10
OSA	‐	‐	‐	‐	‐	−0.11	0.92	−0.45	−0.01	0.11	0.04
NCDS	‐	‐	‐	‐	‐	‐	0.10	0.07	−0.14	0.77	0.19
OSH	‐	‐	‐	‐	‐	‐	‐	−0.49	−0.25	0.27	0.05
NSA	‐	‐	‐	‐	‐	‐	‐	‐	0.26	0.10	0.42
ATA	‐	‐	‐	‐	‐	‐	‐	‐	‐	0.17	−0.13
NCHD	‐	‐	‐	‐	‐	‐	‐	‐	‐	‐	0.02

ATA, anteversion angle; FHD, femoral head diameter; GTH, greater trochanter height; NCDF, distance between the FHC and a plane parallel to the frontal plane containing the projection of the FHC to the FNA; NCHD, distance between the FHC and a plane parallel to the frontal plane containing the projection of the FHC to the FNA; NCVD, vertical distance between the FHC and a plane parallel to transversal plane containing the projection of the FHC to the FNA; NCVD, vertical distance between the FHC and a plane parallel to transversal plane containing the projection of the FHC to the FNA; NSA, neck‐shaft angle; OSA, absolute offset as the distance between FHC and femoral shaft axis; OSH, horizontal offset; OSV, vertical offset; TFL, total femur length

## Discussion

Femoral morphology is associated with pathology of diseases or incidence of fractures^12^. In Table 3, we summarize the clinical/biomechanical relevance of the parameters in biomechanical prosthetics design and orthopedic surgery planning. The studies performed on European samples report the value of 40 to 46.8 mm for FHD^24^, which is consistent with our FHD measurements (46.29 ± 4.02 mm). Head components that are too small may thus cause impingement, dislocation or mechanical failure^25^. Aberrancy of femoral head shape, known as CAM deformity, is associated with dysmorphisms of the head–neck junction. The main reason for this is the fact that pre‐epiphyseal fusion may be a censorious interval of vulnerability for development of morphologic abnormalities of the femoral head–neck junction. These dysmorphisms, mainly in young adults, should be, therefore, reconstructed to normal hip anatomy through surgical therapy (arthroscopic or mini‐open surgery) to avoid the progression of osteoarthritis^25^. The aforementioned examples highlight the importance of considering FHD in biomedical and orthopedic applications. Moreover, because joint ROM is also highly dependent on head size, FHD becomes essential for prosthetic design.

**Table 3:** Surgical and biomechanical relevance of proximal human femur's morphological parameters

Morphological parameters	Clinical and biomechanical relevance
FHD	Impingement/Prosthetics design/To deal with Cam‐deformity
OSA	To design best‐fit prosthetics/Restoration of physiological hip anatomy during THR
OSV	To design best‐fit prosthetics
OSH	To design best‐fit prosthetics
ATA	Preoperative planning of valgus/Varus derotational osteotomy of the proximal femur/To design best‐fit prosthetics
NSA	Better predict hip fracture/Preoperative planning of valgus/Varus derotational osteotomy of the proximal femur
GTH	Restoration of physiological hip anatomy during total hip replacement
TFL	Better prediction of hip fracture
NCDF	Impingement/Hip resurfacing
NCDS	Impingement/Hip resurfacing
NCVD	Impingement/Hip resurfacing
NCHD	Impingement/Hip resurfacing

ATA, anteversion angle; FHD, femoral head diameter; GTH, greater trochanter height; NCDF, distance between the FHC and a plane parallel to the frontal plane containing the projection of the FHC to the FNA; NCHD, distance between the FHC and a plane parallel to the frontal plane containing the projection of the FHC to the FNA; NCVD, vertical distance between the FHC and a plane parallel to transversal plane containing the projection of the FHC to the FNA; NCVD, vertical distance between the FHC and a plane parallel to transversal plane containing the projection of the FHC to the FNA; NSA, neck‐shaft angle; OSA, absolute offset as the distance between FHC and femoral shaft axis; OSH, horizontal offset; OSV, vertical offset; TFL, total femur length

Femoral head offsets (OSA, OSH, and OSV) and anteversion angle (ATA) are required to design best‐fit prosthetics to reduce the incidence of prosthetic failure and loosening^1^. Preoperative knowledge of femoral offset is also essential for THR, because an accurate amount of femoral offset could improve hip abductor strength and enhance joint ROM, while simple restoration promises a reduced risk of wear, dislocation, and failure^1^. Because femoral offset is usually analyzed on radiographs, we measured the OSH as a projection on the frontal plane to mimic this approach (37.90 ± 6.95 mm). The OSA is a direct measurement of the length between head center and FSA perpendicular to the axis of the shaft (42.3 ± 6.0 mm). We measured a value of 54.4 ± 4.1 mm and 7.44 ± 5.1 mm for OSV and GTH, respectively. However, OSV and GTH are poorly presented in the literature. Both OSV and GTH are relevant for restoration of physiological hip anatomy during THR. In most implant designs, a decrease in the offset results in increased instability, which is often counterbalanced using long neck femoral heads. This, in turn, leads to leg length discrepancy. We determined ATA to be 17.5° ± 6.7° in this study. However, ATA reported in the literature largely varies from 10.4° to 24.7° (mean value) in European samples^26^. Presumably, this exists due to different definitions of the axes. Significant variations have been also reported for ATA in right and left hips^27^. Thus far, a satisfactory explanation for this parameter is still lacking. Changes in ATA during childhood play an important role in the physiological development of the hip joint. Malrotation of ATA after intramedullary nailing can cause joint pain, leading to movement limitations, which, therefore, disturbs the patient's daily life. Acceptable preoperative determination of ATA can be directly related to a patient's satisfaction after surgery. ATA is also associated with lower‐extremity disorders, such as in‐toing and out‐toing^28^, impingement, and osteoarthritis^29^. Degenerative diseases of the hip or the knee can accordingly be developed by malrotation. These all highlight the importance of proper orientation of the femoral component in THR to avoid dysfunction.

Good estimation of NSA and TFL can help to better predict hip fracture^30^. Osteotomies of the pelvis and upper femur in surgical management of developmental dysplasia of the hip are very beneficial and enduring. Intertrochanteric osteotomies have been replaced by periacetabular osteotomies for treatment of most dysplasia‐related conditions^31^. NSA and ATA are important parameters required for preoperative planning of valgus or varus derotational osteotomy of the proximal femur. Valgus osteotomy can be useful sometimes to maintain or increase congruency of the hip joint, while varus osteotomy may play a role in optimizing the joint space. A higher NSA relates to the incidence of femoral neck fractures^12^, particularly in osteoporotic patients^32^. A 10° valgus placement of the femoral component can protect against spontaneous fractures of the femoral neck in healthy bone^33^, which underlines the importance of preoperative determination of NSA. The effectiveness of valgus osteotomy (increased NSA) for femoral neck non‐union is unquestioned. Limb‐length discrepancy, malrotations, and posttraumatic deformities can benefit from intertrochanteric osteotomy. Grade II slipped capital femoral epiphysis, Legg‐Calvé‐Perthes disease, and osteonecrosis can sometimes be effectively treated with intertrochanteric osteotomy. Therefore, femoral osteotomies should be thoroughly planned and performed with respect to the possible need for future conversion to total hip replacement.

The parameters NCVD, NCHD, NCDS, and NCDF in addition to FHD play a key role in impingement problems^15^. An adverse relationship between the femoral component size and the neck diameter was found to be a reason for a higher risk of dislocation. Subject to the fact that the femoral component is inserted with strict respect to implantation guidelines, its position should also be in line with the femoral neck axis (FNA). However, coxal anatomy often reveals an offset of the femoral head center (NCVD, NCHD, NCDS, and NCDF), which may have an adverse impact on hip resurfacement dislocation. We, in this study, revealed a relationship between coxa valga/vara and the position of the femoral head center as well as a relationship between anteversion of the neck and position of the femoral head center. These will initially help surgeons to minimize the risk of dislocation in preoperative planning of THR.

In conclusion, this study reports important morphological parameters of the proximal human femur in physiological range to establish a database for orthopedic and biomedical research purposes. The knowledge on the parameter distributions and correlations provide a key tool for further studies, which can contribute to a prolonged lifetime of load‐bearing joint replacements, and reduce the incidence of micro‐motion, impingement, loosening, and periprosthetic fracture.
